# Omega-3 Fatty Acids Prevent Post-Traumatic Stress Disorder-Induced Memory Impairment

**DOI:** 10.3390/biom9030100

**Published:** 2019-03-12

**Authors:** Laiali Alquraan, Karem H. Alzoubi, Hana Hammad, Suzie Y. Rababa’h, Fadia Mayyas

**Affiliations:** 1 Department of Biological Sciences, School of Science, University of Jordan, Amman 11942, Jordan; layaliq@yu.edu.jo (L.A.); hana.hammad@gmail.com (H.H.); Suzie.rababah@bau.edu.jo (S.Y.R.); 2 Department of Biology, Yarmouk University, Irbid 21163, Jordan; 3 Department of Clinical Pharmacy, Jordan University of Science and Technology, Irbid 22110, Jordan; famayyas@just.edu.jo; 4 Department of Medical Science, Irbid Faculty, Al-Balqa Applied University, Irbid 21110, Jordan

**Keywords:** omega-3 fatty acids, post-traumatic traumatic stress, memory, single-prolonged stress, learning, oxidative stress, maze

## Abstract

Post-traumatic stress disorder (PTSD) is a psychiatric disorder that can happen after exposure to a traumatic event. Post-traumatic stress disorder is common among mental health disorders that include mood and anxiety disorders. Omega-3 fatty acids (OMGs) are essential for the maintenance of brain function and prevention of cognition dysfunctions. However, the possible effect of OMG on memory impairment induced by PTSD has not been studied. In here, such an effect was explored using a rat model of PTSD. The PTSD-like behavior was induced in animals using a single-prolonged stress (SPS) rat model of PTSD (2 h restraint, 20 min forced swimming, 15 min rest, 1–2 min diethyl ether exposure). The OMG was administered orally at a dose of 100 mg omega-3 polyunsaturated fatty acid (PUFA)/100 g body weight/day. Spatial learning and memory were assessed using the radial arm water maze (RAWM) method. Changes in oxidative stress biomarkers, thiobarbituric acid reactive substances (TBARS), and brain derived neuroptrophic factor (BDNF) in the hippocampus following treatments were measured. The results revealed that SPS impaired both short- and long-term memory (*p* < 0.05). Use of OMG prevented memory impairment induced by SPS. Furthermore, OMG normalized SPS induced changes in the hippocampus that reduced glutathione (GSH), oxidized glutathione (GSSG), GSH/GSSG ratios, the activity of catalase, glutathione peroxidase (GPx), and TBARSs levels. In conclusion, the SPS model of PTSD-like behavior generated memory impairment, whereas OMG prevented this impairment, possibly through normalizing antioxidant mechanisms in the hippocampus.

## 1. Introduction

Post-traumatic stress disorder (PTSD) is a psychiatric disorder that occurs after exposure to a traumatic event. It involves both mood and anxiety disorders [1,2]. Approximately 20% of individuals exposed to a significant traumatic event will develop PTSD, and children may be at an even higher risk [3]. Post-traumatic stress disorder prevalence rates are largely similar across countries, with the highest rates being found in post-conflict settings [4].

Oxidative stress has been implicated in the response to stress and in the pathogenesis of neurologic and psychiatric diseases [5]. Post-traumatic stress disorder constitutes a form of persistent life stress and is associated with increased oxidative stress and accelerated cellular aging. Clinical and structural neuroimaging studies have repeatedly found associations between PTSD and loss of neural integrity in the hippocampus, amygdala, medial prefrontal, and anterior cingulate cortices [6,7]. While several drugs and psychotherapies are used to treat PTSD, yet there is not enough reliable evidence to draw conclusions about the effectiveness of most treatments [8,9].

The value of nutritional supplements for the treatment of mental disorders stems from studying the association of some food deficiencies with mental disorders. The omega polyunsaturated fatty acids are categorized into n-3 (or omega-3) and n-6 (or omega-6) groups. Eicosapentaenoic acid (EPA) and docosahexaenoic acid (DHA), the main bioactive constituents of omega-3, are not efficiently produced in humans and should consequently be taken directly from the diet, mainly by consuming fish [10]. The omega-3 fatty acids are important because they are essential for the maintenance and function of the brain in addition to cognition. Brain phospholipids are important for intact memory and cognitive functions [11]. In addition, neurodegenerative disease features a cognitive decrease paralleled by an insufficiency in blood and brain levels of DHA [12]. Dietary omega-3 fatty acid supplements enhance cognitive function, promote neuroplasticity, and improve neurological lesion. 

Animal model for PTSD, namely the single-prolonged stress (SPS) model in rats, are very well established and widely used to study PTSD-like behaviors [13,14,15,16,17,18,19,20]. Moreover, omega-3 is well-absorbed in the rat gastrointestinal (GI) tract, where it is distributed to brain via the blood brain barrier [21,22,23,24]. In this study, we investigated the possible protective effect of omega-3 fatty acid on learning ability and memory functions in a rat model of PTSD-like behavior. In addition, the effect of omega-3 on oxidative stress biomarkers was investigated. 

## 2. Materials and Methods 

### 2.1. Animals and Treatment

Male Wister rats weighing 150–200 g (age: 8–10 weeks) were obtained from the animal facility at Jordan University of Science and Technology (JUST) and were used in this study. Animals were housed in plastic cages (five animals per cage) under hygienic conditions in a climate-controlled room (24 ± 1 °C) with at libitum access to rat chow and water. Rats were identified by tail labelling. They were housed under a 12 h light/dark cycle (light on: 7:00 am). All experimental procedures were applied during the light cycle. The protocol of the study was approved by the Institutional Animal Care and Use Committee of Jordan University of Science and Technology (16/3/3/170).

Animals were randomly assigned into four groups (15 rats/group): control, omega-3 fatty acids (OMG), SPS, and OMG/SPS. Animals were acclimatized for one week before the experimental manipulations were started. The PTSD and PTSD + OMG groups were subjected to the SPS model of PTSD on the first day of experiments. At the same day, the OMG and PTSD + OMG groups were started on Menhaden fish oil (24.3% EPA and DHA, Sigma-Aldrich, St. Louis, MI, USA) at a dose of 4 µL/g body weight (100 mg omega-3 polyunsaturated fatty acid (PUFA)/100 g body weight (BWT)) via oral gavage daily for 6 days per week for a total of four weeks and during the radial arm water maze (RAWM) testing day. Concurrently, the control and PTSD groups were administered corn oil via oral gavage once daily. The control group was not exposed to the SPS procedure. For a particular animal, the RAWM testing was carried out on the next day right after the end of the four weeks period.

### 2.2. Induction of Single-Prolonged Stress Model 

The SPS, which is a well-established model for PTSD-like behavior [13,25,26,27,28], was applied to animals of the PTSD and PTSD + OMG groups. Each animal was placed in a double layered plastic Ziploc bag and covered by duct tape in order to ensure complete immobilization for two hours. This was followed by forced swimming for 20 min in a transparent cylindrical container (50 cm in height; 35 cm in diameter; 35 cm water depth). After that, each animal was placed in a cage for 15 min, followed by ether anesthesia for 1–2 min until loss of consciousness.

### 2.3. Radial Arm Water Maze 

The RAWM was used to test spatial learning and memory [13,29,30,31]. The RAWM is a standard model for testing of hippocampus-dependent learning and memory, and it has been extensively used as a single test for that purpose [32,33,34,35,36,37]. It consists of six arms radiating out to an open central area to form six swimming paths with an escape platform located at the end of a goal arm that is kept constant for each particular rat during all trials/tests with different starting arm at each trial/test. All four groups were tested using the RAWM for spatial learning ability and memory performance after completing the 21-day treatment period. All experiments were carried out in a dimly lit room with visual cues fixed on the walls of the room during the experiment. Water temperature was maintained at 23 ± 1 °C. Each animal had to find the submerged platform (2 cm beneath water level) located at the end of the one swimming arm (goal arm) in one minute. There were two phases: the learning phase and the testing phase. The learning phase consisted of two sessions; each session had six trials, one minute/trial, and five minutes of rest between the two sessions. During each trial, the animal was allowed to freely swim to find the submerged platform. However, it was guided to the platform after spending one minute of swimming without finding hidden platform. Once on the platform, the animal was left there for 15 s to observe visual cues on the walls before the next trial was started. In memory tests, the animal was neither guided to the platform nor given 15 s on the platform. Each rat had to undergo 12 learning trials followed by three memory tests. The short-term memory test, which was done 30 min after the last learning trial, and the long-term memory tests, which were done 5 h and 24 h after the last learning trial. In the memory tests, each rat was given one minute to locate the hidden platform. An error was recorded when the rat entered to any arm other than the goal arm. 

### 2.4. Brain Dissection 

Animals were killed by decapitation, and the brain was immediately dissected out. The brain was then placed over filter paper impregnated with normal saline over a cold glass dish filled with crushed ice. The hippocampus was isolated and placed immediately in a previously labeled Eppendorf tube and transferred to a container filled with liquid nitrogen. Eppendorf tubes were placed at −70 °C until analysis.

### 2.5. Molecular Assays 

The obtained hippocampus tissues were homogenized using 200 μL of homogenization buffer prepared by reconstitution of one tablet of phosphate buffered saline (Sigma Chemical CO., Saint Louis, MO, USA) and two protease inhibitor tablets (Sigma Chemical CO.) in 200 mL of distilled water using a plastic pestle [38]. The homogenized tissues were centrifuged at 15,000× *g* for 10 min at 4 °C in order to remove insoluble materials. The supernatant was obtained and stored for further analysis. Total protein concentration in the obtained supernatant was estimated using an available commercial kit (Bio-Rad, Hercules, CA, USA). 

To quantify total glutathione, tissues homogenates were deproteinized with 5% of 5-sulfosalicylic acid (SSA) solution, centrifuged at 10,000× *g* for 10 min at 4 °C to remove the precipitated protein, and then assayed photometrically for glutathione according to the kit’s instructions (Glutathione assay kit, Sigma-Aldrich, St. Louis, MI, USA). For oxidized glutathione (GSSG) measurement, 10 μL of 1M 2-vinylpyridine (Glutathione assay kit, Sigma-Aldrich) was added per 1 mL of supernatant of the sample, then the procedure was carried out as described above for total glutathione. The GSH was then calculated by subtracting the GSSG value from total glutathione. Glutathione peroxidase (GPx) activity was determined using a cellular activity assay kit (CGP1, Sigma-Aldrich). Catalase and superoxide dismutase (SOD) activities were measured using commercially available kits according to manufacturer instructions (SOD: Sigma-Aldrich Corp; Catalase: Cayman Chem, Ann Arbor, MI, USA). Thiobarbituric acid reactive substance (TBARS) level in the hippocampus homogenized tissue was measured using a TBARS assay kit (Cayman Chem. Com., Ann Arbor, MI, USA). Plates were read at the kit’s specified wavelengths using an automated reader (Epoch Microplate Spectrophotometer, Bio-tek instruments, Highland Park, Winooski, VT, USA). 

### 2.6. Statistical Analysis 

All statistics were carried out using the GraphPad Prism (4.0) computer program (GraphPad Software, San Diego, CA, USA). Three-way analysis of variance (ANOVA) was used to compare the number of errors in RAWM learning trials with a multiple comparison post-test. Time (repeated measures factor) and interaction with omega-3/no omega-3 and PTSD/no PTSD were used as independent group dimensions. Comparisons of the number of errors in RAWM memory tests and immunoassays results were carried out using two-way ANOVA followed by Tukey’s post-test. *p* < 0.05 was considered statistically significant. All values are represented as mean ± standard error of the mean (SEM).

## 3. Results

### 3.1. Effect of Post-Traumatic Stress Disorder and/or Omega-3 on Learning and Memory 

**Figure 1:**
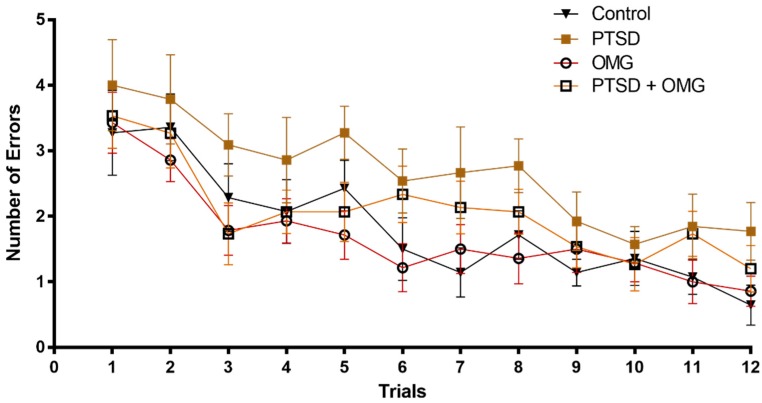
Omega-3 and/or single-prolonged stress (SPS) model of post-traumatic stress disorder (PTSD) did not affect learning performance. The number of errors made by each animal decreased with continued learning trials (1 through 12) without significant differences among control, PTSD, omega-3 treated animals (OMG), and post-traumatic stress disorder (PTSD) treated with OMG (PTSD + OMG) groups. Each point is the mean ± standard error of the mean (SEM) of 15 rats.

In the acquisition phase, rats from all groups (Control, OMG, PTSD, PTSD + OMG) recorded high a number of errors, and then, gradually, the number of errors was reduced as the animals did more trials. No significant differences in RAWM performance were observed among all experimental groups (Figure 1). As learning trials continued, the number of errors started to gradually decrease with no observed significant interaction among treatment groups (Time: *F*_(11,672)_ = 11.85, *p* < 0.001, all other main effects and interactions were non-significant (*p* > 0.05), Figure 1).

**Figure 2:**
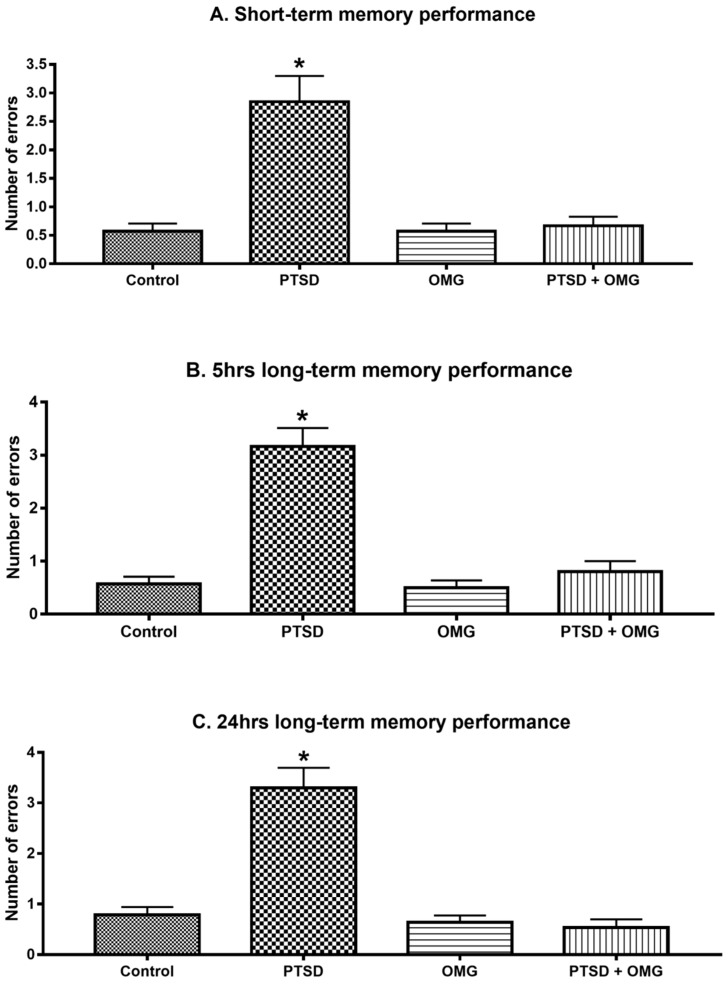
Omega-3 prevents short- and long-term memory impairment induced by the SPS model of PTSD. (**A**) Short-term memory test (trial after 30 min), (**B**) and (**C**) long-term memory tests after 5 h and 24 h, respectively, among control, PTSD, OMG, and PTSD treated with OMG (PTSD + OMG) groups. * Indicate significant difference from all other groups (*p* < 0.05). Each point is the mean ± SEM of 15 animals.

In all short-term (30 min) and long-term (5 h and 24 h) memory tests, significant differences were detected between OMG treated and non-OMG treated groups, and/or PTSD versus non-PTSD groups. Additionally, the presence of both factors, OMG and PTSD, resulted in significant interactions in all memory tests (short-term memory test: *F*(_1,56)_ = 19.25, *p* < 0.001, Figure 2A; long-term 5 h memory test: *F*_(1,56)_ = 29.46, *p* < 0.001, Figure 2B; long-term 24 h memory test: *F*_(1,56)_ = 41.17, *p* < 0.001, Figure 2C).

### 3.2. Effect of Post-Traumatic Stress Disorder and/or Omega-3 on Hippocampus Oxidative Stress Markers

**Figure 3:**
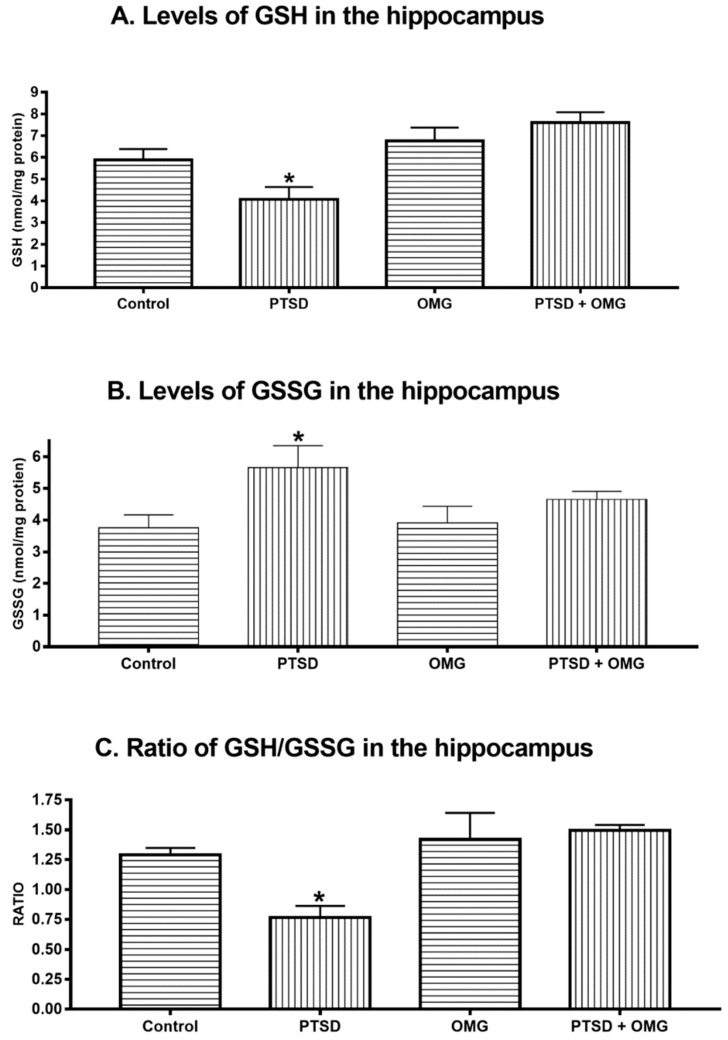
Effect of omga-3 and/or SPS model of PTSD on the levels of glutathione (GSH), oxidized glutathione (GSSG), and GSH/GSSG ratio in the hippocampus. (**A**) Hippocampal GSH levels, (**B**) hippocampal GSSG levels, and (**C**) hippocampal GSH/GSSG ratio. Among control, post-traumatic stress disorder (PTSD), omega-3 treated animals (OMG), and PTSD treated with OMG (PTSD + OMG) groups. * Indicate significant difference relative to other groups (*p* < 0.05). Each point is the mean ± SEM of 15 rats.

**Figure 4:**
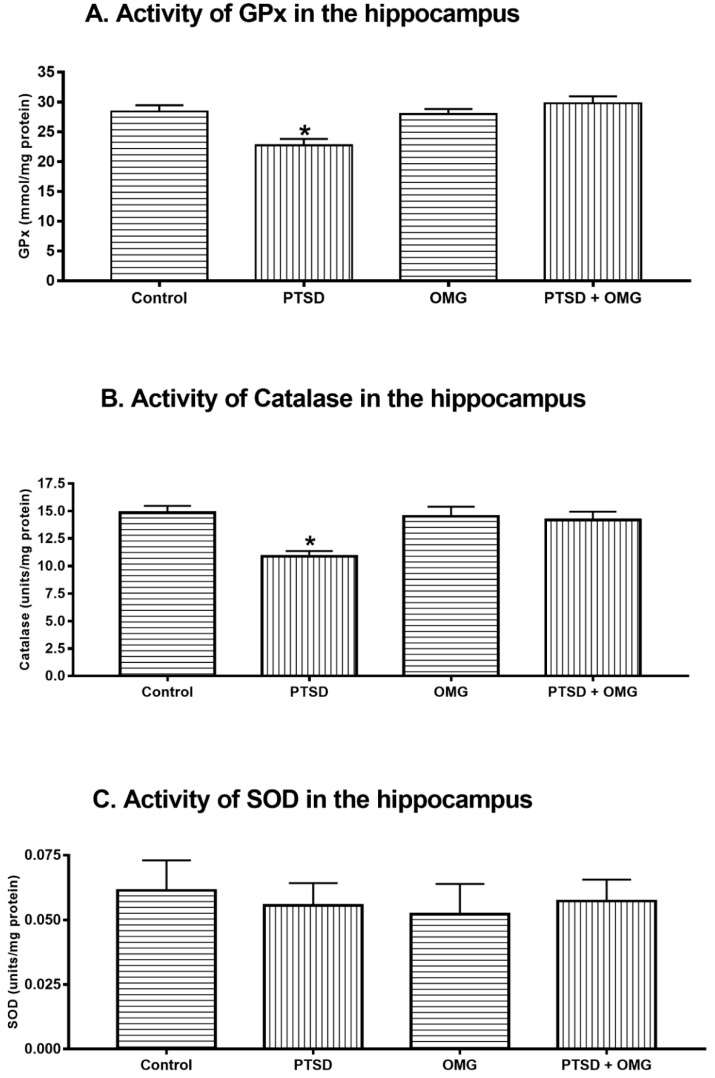
Effect of omega-3 and/or SPS model of PTSD on the activity of anti-oxidative stress enzymes in the hippocampus. (**A**) Hippocampal GPx activity, (**B**) hippocampal catalase activity, and (**C**) hippocampal superoxide dismutase (SOD) activity among control, post-traumatic stress disorder (PTSD), omega-3 treated animals (OMG), and PTSD treated with OMG (PTSD + OMG) groups. * Indicate significant difference relative to other groups (*p* < 0.05). Each point is the mean ± SEM of 15 rats.

There was a significant reduction in the level of GSH in the PTSD group compared to control, OMG, and PTSD + OMG groups (*F*_(1,56)_ = 8.16, *p* < 0.05, Figure 3A). On the other hand, GSSG (the oxidized from of glutathione) was significantly increased (*F*_(1,56)_ = 9.24, *p* < 0.01, *p* ˂ 0.05) in the SPS group compared to other groups (Figure 3B). Additionally, a significant decrease (*F*_(1,56)_ = 13.64, *p* < 0.001) was detected in the GSH/GSSG ratio in the PTSD group compared to other groups. These results indicate that omega-3 treatment prevented alterations in the levels of GSSG and the GSH/GSSG ratio (Figure 3C). The activity of GPx was reduced in the PTSD group compared to the control (*F*_(1,56)_ = 16.16, *p* < 0.001, Figure 4A). Administration of omega-3 prevented SPS induced reduction in GPx activity (*F*_(1,56)_ = 16.16, *p* < 0.001, Figure 4A). The SPS model of PTSD significantly decreased hippocampal catalase activity compared to the control group (*F*_(1,56)_ = 9.09, *p* < 0.01, Figure 4B). Administration of omega-3 prevented the reduction in catalase activity induced by PTSD (Figure 4B). There was no significant difference (*p* < 0.05) detected in the activity of SOD in the hippocampus among all experimental groups (*F*_(1,56)_ = 0.36, *p* > 0.05, Figure 4C).

### 3.3. Effect of Post-Traumatic Stress Disorder and/or Omega-3 on the Hippocampal Thiobarbituric Acid Reactive Species 

**Figure 5:**
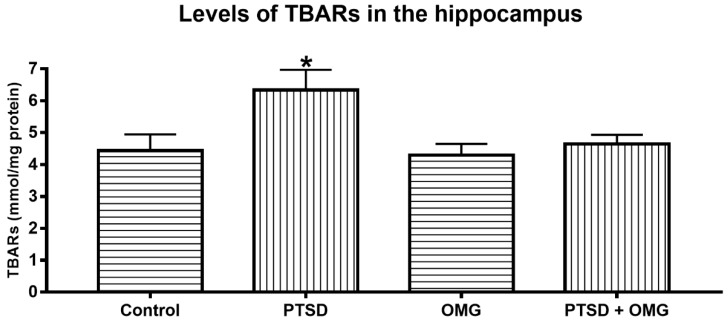
Changes in hippocampal thiobarbituric acid reactive substance (TBARS) levels: Levels of TBARS were significantly increased in post-traumatic stress disorder (PTSD) compared to other experimental groups. Moreover, the levels of TBARS were similar in the omega-3 treated (OMG), PTSD treated with OMG (PTSD + OMG), and control groups. * Indicate significant difference relative to control (*p* < 0.05). Each point is the mean ± SEM of 15 rats.

The SPS significantly increased TBARS levels when compared to other groups (*F*_(1,56)_ = 7.80, *p* < 0.05). Omega-3 administration normalized this elevation, as shown in Figure 5. 

## 4. Discussion

The present study aims to identify the possible protective effects of omega-3 fatty acid supplementation on improving learning ability, enhancing short- and long-term memory, and reducing oxidative stress levels in an SPS rat model. The SPS is a rodent model of traumatic stress exposure that produces key PTSD phenotypes such as impairment in both learning and memory [15,25,39,40,41]. Current results are consistent with previous findings that examined short- and long-term memory impairments in rats exposed to SPS as a model of PTSD [40,42,43]. Administration of omega-3 prevented both short- and long-term memory impairment in OMG/PTSD. Current findings are in agreement with results of other studies that examined the protective effects of omega-3 on cognitive impairment associated with different conditions [44,45]. 

In rat model of traumatic brain injury [46], memory impairments were associated with significant declines in antioxidant mechanisms. Similarly, current results showed that SPS significantly decreased hippocampal antioxidants, mainly GSH and the GSH/GSSG ratio, and activities of GPx and catalase. These findings suggest that oxidative stress leads to brain neuronal damage and memory impairment. Several studies link oxidative stress to cognitive impairments in a number of health conditions, such as in rat model of social stress [47], sporadic dementia of Alzheimer’s type [48], sleep deprivation [49], consumption of high-fat diet [29], and PTSD [13,25]. Interestingly, we show that treatment with omega-3 prevented PTSD-induced cognitive impairments and reduced oxidative stress biomarkers. These findings are consistent with findings of previous studies [13,25]. It has been found that omega-3 enriched dietary supplements attenuate traumatic brain injury in rats and reduce plasticity and learning ability impairment [46]. In addition, omega-3 was shown to reduce the risk of age-related brain impairments [50], improve cognition in older adult women [51], facilitate functional recovery after ischemic brain damage [44], and prevent post-traumatic distress after accidental injury [52]. The PTSD was also revealed to impair memory by increasing oxidant biomarkers such as GSSG level in the hippocampus tissue. Current results are consistent with our previous studies in PTSD rat model [25,41]. 

Thiobarbituric acid reactive substances are byproducts of lipid peroxidation and markers of oxidative stress. Following spatial learning and memory tests, hippocampal levels of TBARS were significantly increased compared to other groups. Notably, high TBARS level were also reported in the cerebral cortex and hippocampus of animals exposed to restraint stress [53,54]. Interestingly, omega-3 prevented TBARS elevations, which is consistent with decreased plasma TBARS following omega-3 supplementation in rats [55]. In addition, omega-3 induced a reduction of TBARS levels in rats’ corpus stratium [56]. On other hand, no differences in plasma TBARS were observed after fish oil supplementation in a rat model of streptozotocin induced diabetes [57].

In conclusion, this study shows a protective effect of omega-3 fatty acid against PTSD-induced short- and long-term memory impairment, possibly through preventing alterations in oxidative stress biomarkers in the hippocampus of PTSD animals.

## References

[1] Lakhan S.E., Vieira K.F. (2008). Nutritional therapies for mental disorders. Nutr. J..

[2] Resick P.A., Monson C.M., Rizvi S.L. (2013). Posttraumatic stress disorder. Psychopathology: History, Diagnosis, and Empirical Foundations.

[3] Peterson K.C., Prout M.F., Schwarz R.A. (2013). Post-Traumatic Stress Disorder: A Clinician’s Guide.

[4] Atwoli L., Stein D.J., Koenen K.C., McLaughlin K.A. (2015). Epidemiology of posttraumatic stress disorder: Prevalence, correlates and consequences. Curr. Opin. Psychiatry.

[5] Sorce S., Krause K.-H. (2009). NOX enzymes in the central nervous system: From signaling to disease. Antioxid. Redox Signal..

[6] Kühn S., Gallinat J. (2013). Gray matter correlates of posttraumatic stress disorder: A quantitative meta-analysis. Biol. Psychiatry.

[7] Pitman R.K., Rasmusson A.M., Koenen K.C., Shin L.M., Orr S.P., Gilbertson M.W., Milad M.R., Liberzon I. (2012). Biological studies of post-traumatic stress disorder. Nat. Rev. Neurosci..

[8] Becker C.B., Zayfert C., Anderson E. (2004). A survey of psychologists’ attitudes towards and utilization of exposure therapy for PTSD. Behav. Res. Ther..

[9] Ghaffarzadegan N., Larson R.C. (2015). Postraumatic Stress Disorder: Five Vicious Cycles that Inhibit Effective Treatment. US Army Med. Dep. J..

[10] Bourre J.-M. (2005). Dietary omega-3 fatty acids and psychiatry: Mood, behaviour, stress, depression, dementia and aging. J. Nutr. Health Aging.

[11] Glade M.J., Smith K. (2015). Phosphatidylserine and the human brain. Nutrition.

[12] Belkouch M., Hachem M., Elgot A., Van A.L., Picq M., Guichardant M., Lagarde M., Bernoud-Hubac N. (2016). The pleiotropic effects of omega-3 docosahexaenoic acid on the hallmarks of Alzheimer’s disease. J. Nutr. Biochem..

[13] Alzoubi K.H., al Subeh Z.Y., Khabour O.F. (2017). Evaluating the protective effect of etazolate on memory impairment, anxiety- and depression-like behaviors induced by post traumatic stress disorder. Brain Res. Bull..

[14] Alzoubi K.H., Al-Ibbini A.M., Nuseir K.Q. (2018). Prevention of memory impairment induced by post-traumatic stress disorder by cerebrolysin. Psychiatry Res..

[15] El-Elimat T., Alzoubi K.H., AbuAlSamen M.M., al Subeh Z.Y., Graf T.N., Oberlies N.H. (2019). Silymarin Prevents Memory Impairments, Anxiety, and Depressive-Like Symptoms in a Rat Model of Post-Traumatic Stress Disorder. Planta Med..

[16] Chen C.V., Chaby L.E., Nazeer S., Liberzon I. (2018). Effects of Trauma in Adulthood and Adolescence on Fear Extinction and Extinction Retention: Advancing Animal Models of Posttraumatic Stress Disorder. Front. Behav. Neurosci..

[17] Lin C.C., Chang H.A., Tai Y.M., Chen T.Y., Wan F.J., Chang C.C., Tung C.S., Liu Y.P. (2019). Subchronic administration of aripiprazole improves fear extinction retrieval of Pavlovian conditioning paradigm in rats experiencing psychological trauma. Behav. Brain Res..

[18] Liu H., Atrooz F., Salvi A., Salim S. (2017). Behavioral and cognitive impact of early life stress: Insights from an animal model. Prog. Neuropsychopharmacol. Biol. Psychiatry.

[19] Solanki N., Alkadhi I., Atrooz F., Patki G., Salim S. (2015). Grape powder prevents cognitive, behavioral, and biochemical impairments in a rat model of posttraumatic stress disorder. Nutr. Res..

[20] Wang S.C., Lin C.C., Chen C.C., Tzeng N.S., Liu Y.P. (2018). Effects of Oxytocin on Fear Memory and Neuroinflammation in a Rodent Model of Posttraumatic Stress Disorder. Int. J. Mol. Sci..

[21] Chen I.S., Subramaniam S., Cassidy M.M., Sheppard A.J., Vahouny G.V. (1985). Intestinal absorption and lipoprotein transport of (omega-3) eicosapentaenoic acid. J. Nutr..

[22] Filloux F., Karras J., Imperial J.S., Gray W.R., Olivera B.M. (1994). The distribution of omega-conotoxin MVIICnle-binding sites in rat brain measured by autoradiography. Neurosci. Lett..

[23] Salem N.M., Lin Y.H., Moriguchi T., Lim S.Y., Salem N., Hibbeln J.R. (2015). Distribution of omega-6 and omega-3 polyunsaturated fatty acids in the whole rat body and 25 compartments. Prostaglandins Leukot. Essent. Fatty Acids.

[24] Takemura M., Kiyama H., Fukui H., Tohyama M., Wada H. (1989). Distribution of the omega-conotoxin receptor in rat brain. An autoradiographic mapping. Neuroscience.

[25] Alzoubi K.H., Rababa’h A.M., Al Yacoub O.N. (2018). Tempol prevents post-traumatic stress disorder induced memory impairment. Physiol. Behav..

[26] Li X.M., Han F., Liu D.J., Shi Y.X. (2010). Single-prolonged stress induced mitochondrial-dependent apoptosis in hippocampus in the rat model of post-traumatic stress disorder. J. Chem. Neuroanat..

[27] Patki G., Li L., Allam F., Solanki N., Dao A.T., Alkadhi K., Salim S. (2014). Moderate treadmill exercise rescues anxiety and depression-like behavior as well as memory impairment in a rat model of posttraumatic stress disorder. Physiol. Behav..

[28] Yamamoto S., Morinobu S., Takei S., Fuchikami M., Matsuki A., Yamawaki S., Liberzon I. (2009). Single prolonged stress: Toward an animal model of posttraumatic stress disorder. Depress. Anxiety.

[29] Alzoubi K.H., Khabour O.F., Salah H.A., Hasan Z. (2013). Vitamin E prevents high-fat high-carbohydrates diet-induced memory impairment: The role of oxidative stress. Physiol. Behav..

[30] Alzoubi K.H., Mayyas F.A., Mahafzah R., Khabour O.F. (2018). Melatonin prevents memory impairment induced by high-fat diet: Role of oxidative stress. Behav. Brain Res..

[31] Alzoubi K.H., Rawashdeh N.Q., Khabour O.F., El-Elimat T., Albataineh H., Al-Zghool H.M., Alali F.Q. (2017). Evaluation of the effect of *Moringa peregrina* extract on learning and memory: Role of oxidative stress. J. Mol. Neurosci..

[32] Alzoubi K.H., Khabour O.F., Alharahshah E.A., Alhashimi F.H., Shihadeh A., Eissenberg T. (2015). The Effect of Waterpipe Tobacco Smoke Exposure on Learning and Memory Functions in the Rat Model. J. Mol. Neurosci..

[33] Alzoubi K.H., Malkawi B.S., Khabour O.F., El-Elimat T., Alali F.Q. (2018). *Arbutus andrachne* L. Reverses Sleep Deprivation-Induced Memory Impairments in Rats. Mol. Neurobiol..

[34] Alzoubi K.H., Rababa’h A.M., Owaisi A., Khabour O.F. (2017). l-Carnitine prevents memory impairment induced by chronic REM-sleep deprivation. Brain Res. Bull..

[35] Rababa’h A.M., Alzoubi K.H., Atmeh A. (2018). Levosimendan enhances memory through antioxidant effect in rat model: Behavioral and molecular study. Behav. Pharmacol..

[36] Aleisa A.M., Alzoubi K.H., Gerges N.Z., Alkadhi K.A. (2006). Nicotine blocks stress-induced impairment of spatial memory and long-term potentiation of the hippocampal CA1 region. Int. J. Neuropsychopharmacol..

[37] Gerges N.Z., Alzoubi K.H., Park C.R., Diamond D.M., Alkadhi K.A. (2004). Adverse effect of the combination of hypothyroidism and chronic psychosocial stress on hippocampus-dependent memory in rats. Behav. Brain Res..

[38] Mhaidat N.M., Alzoubi K.H., Khabour O.F., Tashtoush N.H., Banihani S.A., Abdul-Razzak K.K. (2015). Exploring the effect of vitamin C on sleep deprivation induced memory impairment. Brain Res. Bull..

[39] Eagle A.L., Fitzpatrick C.J., Perrine S.A. (2013). Single-prolonged stress impairs social and object novelty recognition in rats. Behav. Brain Res..

[40] Alzoubi K.H., Khabour O.F., Ahmed M. (2018). Pentoxifylline prevents post-traumatic stress disorder induced memory impairment. Brain Res. Bull..

[41] Alzoubi K.H., Mokhemer E., Abuirmeileh A.N. (2018). Beneficial effect of etazolate on depression-like behavior and, learning, and memory impairment in a model of Parkinson’s disease. Behav. Brain Res..

[42] Bremner J.D., Scott T.M., Delaney R.C., Southwick S.M., Mason J.W., Johnson D.R., Innis R.B., McCarthy G., Charney D.S. (1993). Deficits in short-term memory in posttraumatic stress disorder. Am. J. Psychiatry.

[43] Whitaker A.M., Gilpin N.W., Edwards S. (2014). Animal models of post-traumatic stress disorder and recent neurobiological insights. Behavioural. Pharmacol..

[44] Fernandes J.S., Mori M.A., Ekuni R., Oliveira R.M., Milani H. (2008). Long-term treatment with fish oil prevents memory impairments but not hippocampal damage in rats subjected to transient, global cerebral ischemia. Nutr. Res..

[45] D’Ascoli T.A., Mursu J., Voutilainen S., Kauhanen J., Tuomainen T.P., Virtanen J.K. (2016). Association between serum long-chain omega-3 polyunsaturated fatty acids and cognitive performance in elderly men and women: The Kuopio Ischaemic Heart Disease Risk Factor Study. Eur. J. Clin. Nutr..

[46] Wu A., Ying Z., Gomez-Pinilla F. (2004). Dietary omega-3 fatty acids normalize BDNF levels, reduce oxidative damage, and counteract learning disability after traumatic brain injury in rats. J. Neurotrauma.

[47] Patki G., Solanki N., Atrooz F., Allam F., Salim S. (2013). Depression, anxiety-like behavior and memory impairment are associated with increased oxidative stress and inflammation in a rat model of social stress. Brain Res..

[48] Javed H., Khan M.M., Ahmad A., Vaibhav K., Ahmad M.E., Khan A., Ashafaq M., Islam F., Siddiqui M.S., Safhi M.M. (2012). Rutin prevents cognitive impairments by ameliorating oxidative stress and neuroinflammation in rat model of sporadic dementia of Alzheimer type. Neuroscience.

[49] Alzoubi K.H., Khabour O.F., Salah H.A., Rashid B.E.A. (2013). The combined effect of sleep deprivation and western diet on spatial learning and memory: Role of BDNF and oxidative stress. J. Mol. Neurosci..

[50] Firlag M., Kamaszewski M., Gaca K., Adamek D., Balasinska B. (2013). The neuroprotective effect of long-term n-3 polyunsaturated fatty acids supplementation in the cerebral cortex and hippocampus of aging rats. Folia Neuropathol..

[51] Johnson E.J., McDonald K., Caldarella S.M., Chung H.Y., Troen A.M., Snodderly D.M. (2008). Cognitive findings of an exploratory trial of docosahexaenoic acid and lutein supplementation in older women. Nutr. Neurosci..

[52] Matsuoka Y., Nishi D., Yonemoto N., Hamazaki K., Hamazaki T., Hashimoto K. (2011). Potential role of brain-derived neurotrophic factor in omega-3 fatty acid supplementation to prevent posttraumatic distress after accidental injury: An open-label pilot study. Psychother. Psychosom..

[53] Budni J., Zomkowski A.D., Engel D., Santos D.B., dos Santos A.A., Moretti M., Valvassori S.S., Ornell F., Quevedo J., Farina M. (2013). Folic acid prevents depressive-like behavior and hippocampal antioxidant imbalance induced by restraint stress in mice. Exp. Neurol..

[54] Fontella F.U., Siqueira I.R., Vasconcellos A.P., Tabajara A.S., Netto C.A., Dalmaz C. (2005). Repeated restraint stress induces oxidative damage in rat hippocampus. Neurochem. Res..

[55] Erdogan H., Fadillioglu E., Ozgocmen S., Sogut S., Ozyurt B., Akyol O., Ardicoglu O. (2004). Effect of fish oil supplementation on plasma oxidant/antioxidant status in rats. Prostaglandins Leukot. Essent. Fatty Acids.

[56] Sarsilmaz M., Songur A., Ozyurt H., Kus I., Ozen O.A., Ozyurt B., Sogut S., Akyol O. (2003). Potential role of dietary omega-3 essential fatty acids on some oxidant/antioxidant parameters in rats’ *corpus striatum*. Prostaglandins Leukot. Essent. Fatty Acids.

[57] Mayyas F., Jaradat R., Alzoubi K.H. (2018). Cardiac effects of fish oil in a rat model of streptozotocin-induced diabetes. Nutr. Metab. Cardiovasc. Dis..

